# Environmental RNA as a Tool for Marine Community Biodiversity Assessments

**DOI:** 10.1038/s41598-022-22198-w

**Published:** 2022-10-22

**Authors:** Marissa S. Giroux, Jay R. Reichman, Troy Langknecht, Robert M. Burgess, Kay T. Ho

**Affiliations:** 1 ORISE C/O US Environmental Protection Agency, Office of Research and Development, Center for Environmental Measurement and Modeling, Atlantic Coastal Environmental Sciences Division, Narragansett, USA; 2 Pacific Ecological Systems Division, US Environmental Protection Agency, Office of Research and Development, Center for Public Health and Environmental Assessment, Corvallis, OR USA; 3 Atlantic Coastal Environmental Sciences Division, US Environmental Protection Agency, Office of Research and Development, Center for Environmental Measurement and Modeling, Narragansett, RI USA

**Keywords:** Marine biology, Molecular ecology, Biodiversity, Sequencing

## Abstract

Microscopic organisms are often overlooked in traditional diversity assessments due to the difficulty of identifying them based on morphology. Metabarcoding is a method for rapidly identifying organisms where Environmental DNA (eDNA) is used as a template. However, legacy DNA is problematically detected from organisms no longer in the environment during sampling. Environmental RNA (eRNA), which is only produced by living organisms, can also be collected from environmental samples and used for metabarcoding. The aim of this study was to determine differences in community composition and diversity between eRNA and eDNA templates for metabarcoding. Using mesocosms containing field-collected communities from an estuary, RNA and DNA were co-extracted from sediment, libraries were prepared for two loci (18S and COI), and sequenced using an Illumina MiSeq. Results show a higher number of unique sequences detected from eRNA in both markers and higher α-diversity compared to eDNA. Significant differences between eRNA and eDNA for all β-diversity metrics were also detected. This study is the first to demonstrate community differences detected with eRNA compared to eDNA from an estuarine system and illustrates the broad applications of eRNA as a tool for assessing benthic community diversity, particularly for environmental conservation and management applications.

## Introduction

Environmental DNA (eDNA) is organismal DNA collected from environmental settings and is a rapidly growing tool with a plethora of monitoring and management applications. However, it is not the only genetic material that is found in environmental samples. RNA is also found alongside DNA in environmental media. Living organisms are constantly producing coding and non-coding RNAs through the process of transcription of DNA templates to maintain homeostasis. As organisms shed DNA through mucous, skin cells, fecal matter, and degrading animal carcasses, RNA is also shed^1,2^. Similarly to eDNA, RNA is found in environmental samples, such as water, soil, sediment, and air, and is termed environmental RNA (eRNA)^3^. Until recently, eDNA has been the primary genetic template of choice for quantitative and qualitative organism identification and community diversity analyses through metabarcoding.

Metabarcoding is a molecular method that allows for the concurrent identification of numerous organisms from metagenomic DNA collected from a single sample. Metabarcoding methods typically utilize eDNA extracted from environmental samples and fecal samples. The DNA template is then PCR amplified for the genetic marker of interest and subsequently sequenced using next-generation sequencing platforms where sequences are matched to taxonomic databases resulting in the rapid genetic identification of organisms^4^. There are regions of several genes that can be sequenced to analyze eukaryotic communities. Most commonly used are mitochondrial Cytochrome Oxidase c subunit 1 (COI) and the nuclear 18S ribosomal RNA (rRNA) gene^5,6^. In marine environments, eDNA barcoding and metabarcoding are powerful tools for mapping marine biodiversity, detecting endangered and invasive species, and assessing community changes to environmental and contaminant stressors^7^. The decreasing costs, sample preparation time, and speed of High Throughput Sequencing compared to traditional biomonitoring methods has contributed to the increased use of eDNA metabarcoding in biodiversity and monitoring surveys. Metabarcoding is a particularly useful tool for the rapid taxonomic identification of organisms that are often difficult and time-consuming to identify using traditional morphological methods^7^. Low sampling frequency and the high cost associated with morphological identification of benthic organisms often results in substantial underestimation of diversity of microscopic organisms whereas molecular methods yield higher taxonomic resolution^8^.

Metabarcoding methods are sensitive enough to detect the presence of microscopic organisms, such as microfauna and meiofauna^9–11^. Meiofauna are characterized as organisms that can pass through a 0.5 mm mesh and are retained by a 45 μM mesh, such as many species of nematodes, copepods, rotifers, and ostracods. Microfauna are classified as < 0.1 mm^12^, such as tardigrades, amoebas, and foraminifera. Due to their small size and the challenges of taxonomic identification, micro- and meiofauna are often discounted in sampling studies. However, meiofauna are vital in functioning food webs, involved in waste removal and biogeochemical cycles, and provide oxygenation to hypoxic areas, thus making marine ecosystems more habitable for other organisms^13–15^. Micro- and meiofauna are sensitive to environmental disturbances, such as pollution and nutrient changes, and are found in both pelagic and benthic aquatic environments, so they serve as ideal biological indicators of healthy ecosystems^16–18^. For example, changes in community structure can help pinpoint specific disturbances and system imbalances. Therefore, metabarcoding is an invaluable tool for rapidly identifying meiofaunal organisms and processing large numbers of samples.

Metabarcoding methods using eDNA have been used to assess benthic communities’ structure and have been validated as a useful approach to characterize benthic meiofaunal communities^10,19^. As such, eDNA and molecular methods of organism detection are being incorporated into aquatic conservation practices through biodiversity surveys, population estimates, biomonitoring of endangered species, and even to predict parasite outbreaks in aquaculture operations^20–23^. Within the United States, eDNA methods are starting to be adopted by federal and local agencies for regulatory and environmental management purposes to supplement traditional monitoring and biodiversity assessment strategies^24^. Increasing the understanding of molecular tools and their practical applications and incorporating appropriate uncertainty into eDNA in management tools will broaden the use of eDNA in decision-making practices^25^.

Although metabarcoding using eDNA has the potential to be an accessible and powerful tool for management applications, DNA has a major downfall when attempting to obtain a snapshot of community structure at a specific timepoint. DNA can be detected from the cells of deceased organisms as well as from cells shed when living organisms pass by sampling sites. This leads to the possibility of an inaccurate representation of the community diversity and composition at the time of sampling. DNA degredation depends on a multitude of environmental factors, including temperature, salinity, pH, UV exposure, bacterial decomposition, and nuclease presence^26^. DNA is relatively stable compared to RNA, particularly in aquatic environments, which increases the possibility of detecting previously deceased organisms in the sample^2^. This is especially important when understanding community composition prior to and in response to disturbance events. Conversely, RNA is predominately expressed by only the living organisms present and it is rapidly degraded in many environments. RNA is much less stable under environmental conditions due to the molecular composition of ribose, which has a hydroxyl group susceptible to hydrolysis, versus deoxyribose. eRNA decay rates are significantly higher compared to eDNA rates, and eRNA half-lives can be 5 h faster than eDNA in aquatic systems^27^. eDNA is degradation is also lower in sediment compared to aqueous samples, so eDNA is more persistent in benthic environments than pelagic ones^28^. Additionally, the ubiquitous presence of RNases in the environment can also quickly decrease RNA integrity. Therefore, using eDNA results in a convoluted representation of past and present organisms while RNA methods show only the living organisms, which is a more accurate measurement of the current state of a community.

RNA and DNA can be successfully co-extracted from samples for use in the metabarcoding pipeline^29^. Then, the RNA of the common barcoding markers, such as COI and 18S, is reversed transcribed into cDNA that can be PCR amplified. Selecting the appropriate loci, or marker gene of interest, and associated primers for PCR amplification is important for the successful detection of species within target communities. In our current study, we investigated marine benthic communities and were specifically targeting meio- and microfaunal communities rather than macrofaunal, bacterial, or algal communities. Throughout the past decade, several new primers have become available for detection of specific communities. Zhang et al. (2018) compared the utility of various primer pairs for different loci and found that using two markers allows for a more comprehensive overview of the species present in the community of interest, particularly when the markers are evolutionarily independent, such as 18S and COI^6^. Additionally, many metabarcoding studies on benthic communities use two primer pairs to maximize taxa detection^30^. Therefore, we selected the Uni18S (V4 region of 18S) and Leray COI primer pairs. These specific primers were selected because they resulted in high read abundance in a study comparing the ability of several metabarcoding primers to detect mock invertebrate communities^6^. The Leray mitochondrial COI DNA primers were also specifically designed to detect marine metazoa, which is also a target community of the current study^31^.

The objective of this study was to compare the detected community composition and diversity between eRNA and eDNA. The current study focuses on optimizing eRNA and eDNA methods for the purpose of evaluating community diversity specifically in meiofaunal and microfaunal communities because of the microscopic size and difficulty in morphological identification of these organisms. Comparing metabarcoding approaches to traditional morphological identification of time-consuming microscope-based counting/identifying has been previously conducted in the same estuary^10^ and is outside of the scope of this study.

## Methods

### Sediment core collection and mesocosm setup

Seven sediment cores were collected from the Narrow River Estuary (RI) during low tide in an area clear of benthic macrofauna growth (41° 26.877ʹ N, − 71° 27.528 ʹ W) (Fig. S1). This location has been used in previous studies for sediment core/ mesocosm collection because it is relatively unimpacted^10,32^. Clear PVC tubes (5 cm outer diameter by 25 cm length) were used for coring and were used as mesocosms representative of the sediment environment of the estuary. Seven cores were collected in total as replicates. Approximately 10 cm of sediment was collected in each mesocosm, capped on both ends with clean rubber stoppers, and immediately transported to the laboratory.

### Mesocosm maintenance and experimental design

Core collection, mesocosm maintenance, and care were based on previous mesocosm studies conducted at the U.S. EPA laboratory in Narragansett, RI (USA)^10^. Mesocosms were arranged vertically in plexiglass holders in flow-through tables at 18.3 ± 0.25 °C on 12 h light: 12 h dark cycles (Fig. S1). Individual air and water lines with fresh, natural seawater (30.33 ± 0.24 ‰) filtered at 5 μm were adjusted to flow at 3–5 ml/min resulting in approximately 8.5–14 turnovers every 24 h in each mesocosm, which were representative of environmental conditions at the time of collection. Water quality measurements, dissolved oxygen, pH, salinity, and temperature were recorded in each mesocosm once a week. Individual flow rate measurements were recorded every two days and adjusted accordingly. To provide feedstocks for the micro/meiofauna in the mesocosms, an equal mixture of 6.7 × 10^6^ cells/species (total 20.1 × 10^6^ cells) consisting of 3 green algae species, *Dunaliella salina, Pavlova lutheri*, and *Tetraselmis spp.*, was added to each mesocosm every other day. Algae was prepared in rotating stocks and algae concentration was determined by counting cells with a hemocytometer prior to feedings. Mesocosms were held in the laboratory for 2 weeks to allow acclimation to artificial conditions and to allow for recovery from mechanical disturbances from the field collection process prior to sampling.

### Nucleic acid collection

After two weeks, the top 1 cm of sediment was collected by gently pouring off overlying water until approximately 1–2 mm of muddy, overlying water remained. Many meiofauna were present in the residual overlying water, so this was collected with the top 1 cm of sediment. A clean, plastic core extruder was used to gently push the top 1.5 cm of sediment out of the PVC tube. Sediment was collected in sterile weigh boats and briefly and gently homogenized with a sterile metal spatula before being stored in two RNAse/DNase-free 15 mL conical tubes per mesocosm for technical replicates. Sediment was then immediately flash frozen and stored at − 80 °C.

RNA and DNA were co-isolated using the Qiagen RNeasy PowerSoil Total RNA Kit followed immediately by the Qiagen RNeasy PowerSoil DNA Elution Kit. Phenol/ chloroform/ isoamyl alcohol 25:24:1 at pH 6.6 and 200 proof Molecular Grade Ethanol used for nucleic acid extraction and PCR cleanup (“PCR cleanup and library preparation” Section) were from Thermo Fisher. Nucleic acids were extracted according to manufacturer instructions, and 2 g of sediment were used in each extraction. There were 7 mesocosm samples and 2 technical replicates extracted per mesocosm. DNA was subsequently cleaned of residual RNA using Qiagen’s RNase A protocol. Briefly, digestion buffer and 0.5 µL of RNase A (Qiagen) was added to each sample and incubated for 40 min at 37 °C. DNA was separated using 200 µL of phenol / chloroform / isoamyl alcohol by centrifugation at 10,000 g for 5 min. The aqueous layer was removed and treated with 5 M NaCl and 100% ethanol. DNA precipitation occurred after a 30 min incubation at − 20 °C follow by centrifuging at 10,000 g for 10 min. Both RNA and DNA purity and concentration were determined using a NanoDrop 8000 (Thermo Scientific). The 260/280 values were between 1.95–2.2 and 1.7–1.85 for RNA and DNA, respectively. Also, RNA integrity was confirmed with gel electrophoresis by the presence of distinct 18S and 28S bands. Agarose, 50X TAE Buffer, SYBR Safe DNA Gel Stain, and 6X DNA Loading Dye used for gel electrophoresis were from Thermo Fisher. Extracted RNA and DNA samples were stored at − 80 °C.

### Amplification of 18S and COI markers

RNA was reverse transcribed to cDNA using the iScript Select cDNA Synthesis Kit (Bio-Rad) according to manufacturer instructions from 500 ng of total RNA using the random primers provided with the kit. cDNA was stored at − 20 °C until PCR amplification.

Two marker genes were selected for PCR amplification: the V4 region of 18S gene (Uni18S) and Cytochrome Oxidase Subunit I (COI). The 18S primer sequences were the Uni18S primers sourced from Zhan et al.^33^ Forward: 5’-AGGGCAAKYCTGGTGCCAGC- 3’, Reverse: 5’-GRCGGTATCTRATCGYCTT-3’. The COI primer sequences were the Leray primers from Leray et al.^31^ Forward: 5’- GGWACWGGWTGAACWGTWTAYCCYCC -3’, Reverse: 5’- TAAACTTCAGGGTGACCAAAAAATCA -3’. Forward and reverse primers had the following adaptor sequences added for compatibility with the Illumina 16S Metagenomic Sequencing Library Prep protocol: Forward 5’- TCGTCGGCAGCGTCAGATGTGTATAAGAGACAG- 3’ and Reverse 5’-GTCTCGTGGGCTCGGAGATGTGTATAAGAGACAG-3’.

PCR reactions were run in 96-well plates on a Bio-Rad C1000 thermal cycler in 15 µL total reaction volumes which consisted of 7.5 µL of Kapa HiFi HotStart ReadyMix, 4.5 µL of nuclease -free H_2_O, 1 µL of forward primer (5 nM), 1 µL of reverse primer (5 nM), and 25 ng of DNA template. All samples were run in duplicate and amplicons from pairs of technical replicates were pooled after first-round PCR. The thermal cycler protocol for the 18S marker was as follows: 5 min at 95 °C followed by 35 cycles of 30 s at 94 °C, 25 s at 71 °C, and 20 s at 72 °C, followed by 5 min at 72 °C. The thermal cycler protocol for the COI marker was as follows: 5 min at 95 °C followed by 35 cycles of 45 s at 94 °C, 45 s at 57.9 °C, and 45 s at 72 °C, followed by 5 min at 72 °C. Successful PCR amplification was confirmed on a 1.25% gel. PCR thermocycler protocols and primer concentrations were optimized to ensure no secondary product formation and strong amplicon band presence on a gel.

### PCR cleanup and library preparation

PCR cleanup was conducted according to the Illumina 16S Metagenomic Library Prep Guide. Briefly, PCR amplicons were cleaned up with AMPure XP Beads (Beckman Coulter) using multiple 80% ethanol washes. Index PCR was conducted with the Nextera XT Index Kit (Illumina) using 5 µL of the cleaned PCR product. The following thermal cycler protocol was used for index PCR for both amplicons: 3 min at 95 °C followed by 8 cycles of 30 s at 95 °C, 30 s at 55 °C, and 30 s at 72 °C, followed by 5 min at 72 °C. A second cleanup was performed after indexing also using AMPure XP Beads. Libraries were run on a Bioanalyzer (Agilent) and a Qubit (Thermo Fisher) to verify amplicon size distribution and concentration, respectively.

### Sequencing and analysis

Library concentrations were normalized and pooled at 5 µL volumes at 4 nM before denaturing with 5 µL of 0.2 N NaOH. Samples were centrifuged at 280 g for 1 min, and then incubated for 5 min at room temperature. A Hybridization Buffer (Illumina) was added to the denatured libraries to dilute to 4–6 pM and the internal standard, PhiX Control Kit V3 (Illumina), was added. Multiplexed libraries were sequenced on an Illumina MiSeq.

Bioinformatic analysis was conducted using QIIME2 version 2019.10^34^. Samples were demultiplexed and trimmed to 280 bp to maintain a quality threshold of 30 or more (see SI Table 1 for read number per mesocosm). Reads from technical replicates of each mesocosm were merged to increase sampling depth. Sequences were denoised to ASVs using the DADA2 pipeline within QIIME2 software^35^ Then, alpha rarefaction was performed on data from both markers to estimate where saturation of metrics (e.g., observed feature) occurred. To generate the core metrics, COI sequences were sampled to a depth of 50,371 (retained 79.9% features in 100% of the samples). For 18S sequences the depth was 18,223 (retained 69.6% features in 100% of the samples). Rarefaction curves for both makers indicate that the number of observed features plateaued well below the sampling depths chosen (see Fig. S2). Sequences were classified using QIIME2 from the SILVA_132 18S database and the protozoan and invertebrate sequences from the Barcode of Life Database (BoLD) for 18S and COI libraries, respectively. Further identification of unassigned ASVs from the initial classification was performed using the NCBI Basic Local Alignment Search Tool (BLAST). Sequences were clustered into Amplicon Sequence Variants (ASVs) based on classification. Observed ASVs and Shannon Index α-diversity metrics as well as Bray–Curtis and Jaccard’s distance β-diversity metrics were calculated using QIIME2. Bray–Curtis dissimilarity was selected as a β-diversity metric because it is a quantitative measurement that accounts for relative abundance of each ASV. Jaccard’s distance was selected because it is a qualitative measurement that is based on the presence or absence of each ASV.

### Statistical analysis

All statistical analyses were conducted in QIIME2. α-diversity results were analyzed using the Kruskal–Wallis test, and β-diversity results were analyzed using PERMANOVA. *P*-values below 0.05 were considered statistically significant for all diversity metrics. Venn diagrams, α-diversity plots, and β-diversity Principal Coordinates Analysis (PCoA) plots were visualized using the ggplot2 package in R (v. 4.1.0) and RStudio (v. 1.4.1717-3)^36,37^.

## Results

### Sample richness

**Figure 1:**
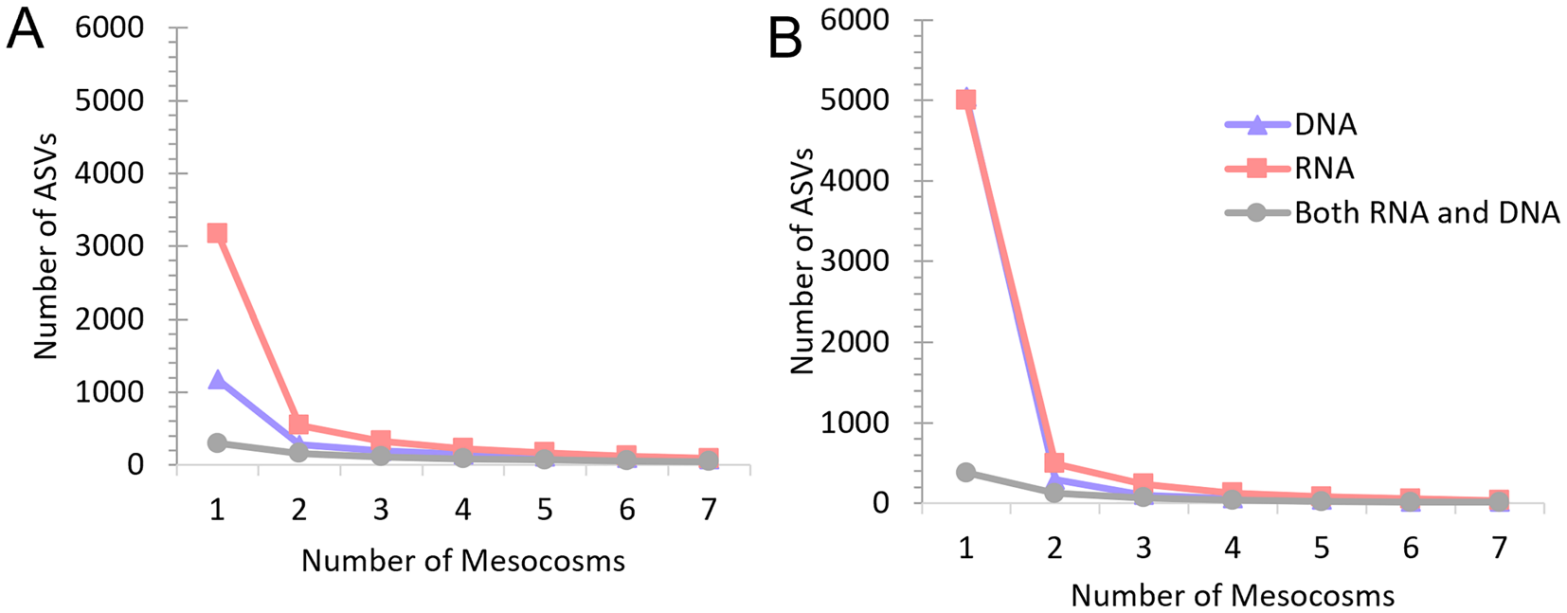
The number of unique sequences (ASVs) detected using the (**A**) 18S marker and (**B**) COI marker for RNA (red), DNA (blue) templates, and found similarly in both RNA and DNA templates (gray) in at least 1, 2, 3, 4, 5, 6, and all 7 of the mesocosms.

Each of the 7 mesocosms were sampled in duplicate for a total of 14 extracted nucleic acid samples, and each sample was sequenced using two markers. To assess the consistency of ASVs detected among individual mesocosms, the common ASVs detected in each number of mesocosms was calculated. For example, an ASV detected in only 1 of the 7 mesocosms may be less representative of the community than ASVs detected in the majority of mesocosms. Figure 1 shows how the number of unique sequences, or amplicon sequence variants (ASVs), detected in at least each number of mesocosms (1–7) differed between the RNA and DNA templates. The number of unique ASVs found within all 7 mesocosms was lower than the number of ASVs detected in just 1 mesocosm, and there was a decrease in number of similar ASVs found in each number of mesocosms for RNA and DNA templates with both markers (Fig. 1A,B). As the number of mesocosms (sampling) increased, the number of ASVs detected in all mesocosms decreased. For both the 18S and COI markers, there were distinct differences between the ASVs detected in the RNA samples compared to the DNA template. Specifically, RNA ASVs for the 18S marker were 2.7-fold higher than DNA ASVs with 297 ASVs detected by both RNA and DNA (Fig. 1A). For the 18S sequences detected in 4 of the mesocosms, the RNA ASVs were approximately 1.6-fold higher than DNA ASVs with 83 ASVs similarly detected by both (Fig. 1A). Similarly, the RNA ASVs detected in 7 mesocosms were 1.2-fold higher than DNA ASVs with 47 ASVs similarly detected with RNA and DNA (Fig. 1A). The number of RNA ASVs detected in at least one mesocosm for the COI marker was approximately the same as DNA ASVs with only 7.6% of the ASVs shared by RNA and DNA (Fig. 1B). However, the RNA ASVs were approximately 2.2 and 2.3-fold higher than the DNA for ASVs detected in 4 and 7 of the mesocosms, respectively (Fig. 1B).

### Taxonomy

**Figure 2:**
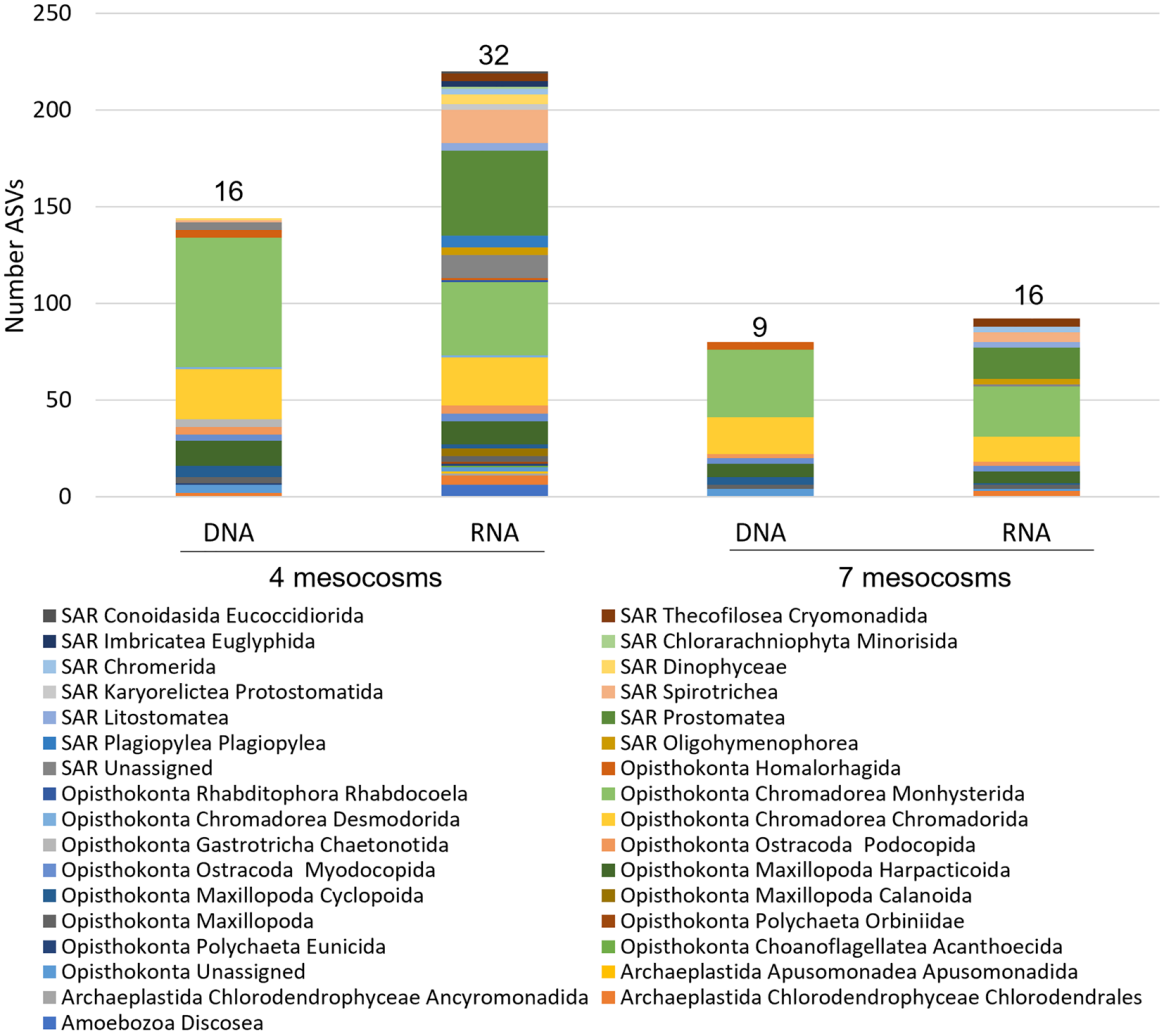
Assigned taxonomy and number of unique sequences (ASVs) detected using the 18S marker for RNA and DNA templates in four and all seven mesocosms. Total number of detected taxa at the Order level for each marker and template stated above the bar. Taxonomy is assigned to Order.

There were 293 unassigned ASVs (0.08%) using DNA and 6560 (1.79%) unassigned ASVs using RNA with the 18S marker using the taxonomic identification from SILVA database. For the COI marker, there were 29,783 (3.38%) unassigned ASVs using DNA and 25,875 (2.93%) unassigned ASVS using RNA using the taxonomic identification with BoLD. ASVs that were not matched to a kingdom were counted as unassigned ASVs. Unique sequences were taxonomically identified to Class and Order based on the presence of each sequence in at least 4 of the mesocosms and additionally categorized based on the presence in all 7 mesocosms. Figure 2 depicts the number of ASVs in each Order and SI Table 2 lists the taxonomic classification and number of unique ASVs for the 18S marker. The 18S marker resulted in 220 unique ASVs for RNA and 144 unique ASVs for DNA for sequences detected in at least 4 of the 7 mesocosms (Fig. 2). There were 92 ASVs for RNA and 80 ASVs for DNA for sequences detected in all 7 of the mesocosms with the 18S marker (Fig. 2). The categorized sequences are also reported as percent of total ASVs (SI Table 2). There were 74 more taxa detected by the RNA template than the DNA template in sequences found in 4 of the mesocosms, and 12 more organisms detected by RNA than DNA in sequences found in all 7 mesocosms. In sequences detected in 4 and 7 mesocosms there were 21 and 10 different classes of organisms detected with the RNA templates, respectively (Fig. 2). With the DNA templates, there were 8 and 4 different classes detected in 4 and 7 mesocosms, respectively (Fig. 2). Chromadorea, a class of nematodes, were the most highly detected class for both RNA and DNA templates with 64 and 97 total ASVs (30% and 66%), respectively, for sequences found in 4 of the 7 mesocosms. Other multicellular organisms detected with both RNA and DNA in 4 of the 7 mesocosms were Polychaeta, Maxillopoda, Ostracoda, and the kinorynch Homalorhagida. Prostomatea, a class of ciliates, was the second highest detected class (20%) for RNA but was not detected in the DNA templates (SI Table 2). There were 6 detected taxa of Amoebozoa Discosea and one Choanoflagellatea Acanthoecida using RNA in 4 of the 7 mesocosms but none detected using DNA. Additionally, no organisms from the Stramenopiles, Alveolates, or Rhizaria (SAR) clade, groups of single cell eukaryotes, were detected with DNA templates in all 7 mesocosms, but there were 12 detected classes within the SAR clade using the RNA template.

**Figure 3:**
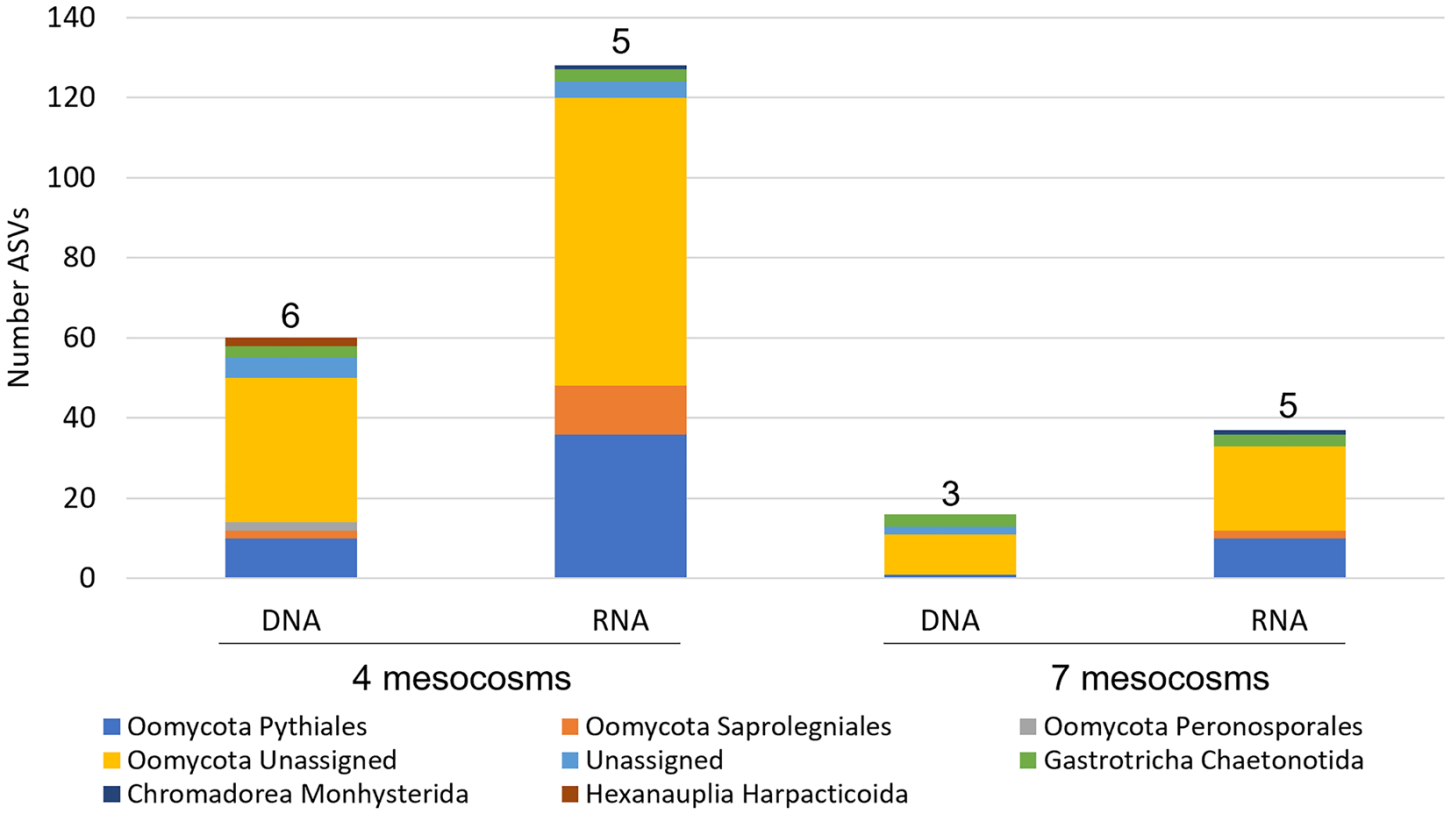
Assigned taxonomy and number of unique sequences (ASVs) detected using the COI marker for RNA and DNA templates in four and all seven mesocosms. Total number of detected taxa at the Order level for each marker and template stated above the bar. Taxonomy is assigned to Order.

The COI marker resulted in 129 unique ASVs in RNA templates and 60 unique ASVs in DNA templates for sequences detected in at least 4 of the 7 mesocosms (Fig. 3). The number of unique ASVs using the COI marker was less than the number of unique ASVs for RNA and DNA templates using the 18S marker. There were 37 ASVs for RNA and 16 ASVs for DNA for sequences detected in all 7 of the mesocosms using the COI marker (Fig. 3). Similarly, total RNA and DNA ASVs for COI were lower than the total ASVs detected using the 18S marker. There were 3 different orders of Oomycota, a phylum within the SAR clade, detected using DNA in 4 of the 7 mesocosms, but only 2 orders detected from RNA (Fig. 3). Pythiales were the most highly detected Oomycota for both RNA and DNA (SI Table 3). Monhysterida, an order of Nematoda, was detected from RNA, but not DNA, using the COI marker. Harpacticoida, an order of copepod, was detected using DNA with the COI marker within 4 of the 7 mesocosms. The Gastrotricha order Chaetonotida was detected using both RNA and DNA in all mesocosms.

The 18S marker detected a richer taxonomic composition compared to the COI marker using RNA where 12 distinct SAR classes were found in 4 of the mesocosms: Oligohymenophorea, Plagiopylea, Prostomatea, Litostomatea, Spirotrichea, Karyorelictea, Dinophyceae, Chromerida, Chlorarachniophyta, Imbricatea, Conoidasida, and Thecofilosea (SI Table 3). The 18S marker was able to detect several ciliates and dinoflagellates in the majority of mesocosms using the RNA marker whereas the COI marker did not. However, the COI marker detected 3 orders of Oomycota (Pythiales, Saprolegniales, and Peronosporales) whereas the 18S marker did not with either the RNA or DNA template. The taxonomic compositions of individual mesocosms for both RNA and DNA templates are displayed as the relative abundance of each taxa in Fig. S3 (18S marker) and Fig. S4 (COI marker).

### Alpha diversity

**Figure 4:**
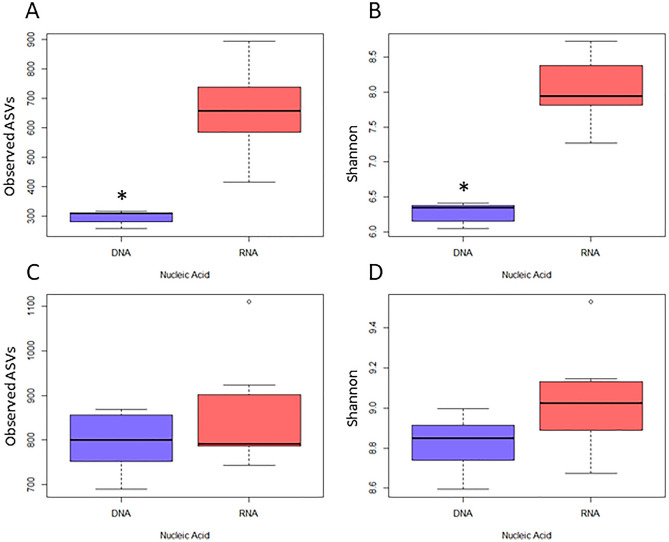
Alpha diversity metrics for DNA (blue) vs. RNA (red) metabarcoding templates for the 18S gene (**A** and **B**) and for the COI gene (**C** and **D**). Bold middle line represents the median ASVs, and upper and lower box limits represent the upper and lower quartiles, respectively. Observed ASVs are reported in A and C, and Shannon Diversity Index scores are reported in B and D. Significant differences (*p* < 0.01) in diversity between DNA and RNA are denoted by an asterisk and were detected using a Kruskal-Wallis test (n = 7).

Alpha diversity metrics are reported as Observed ASVs and as the Shannon Diversity Index. There was a significant difference in the observed ASVs between RNA and DNA templates for the 18S marker (*p* = 0.0017), and the difference in the Shannon Diversity Index was also significant between RNA and DNA templates (*p* = 0.0017) (Fig. 4A,B). The RNA template average observed ASVs and Shannon Diversity Index values were significantly higher than the mean DNA values for the 18 s marker. However, there were no significant differences in either α-diversity index between RNA and DNA in the COI marker (Fig. 4C,D). The COI marker showed a trend towards an increase in higher average RNA Shannon Diversity index values compared to the DNA template (*p* = 0.064) (Fig. 4D).

### Beta diversity

**Figure 5:**
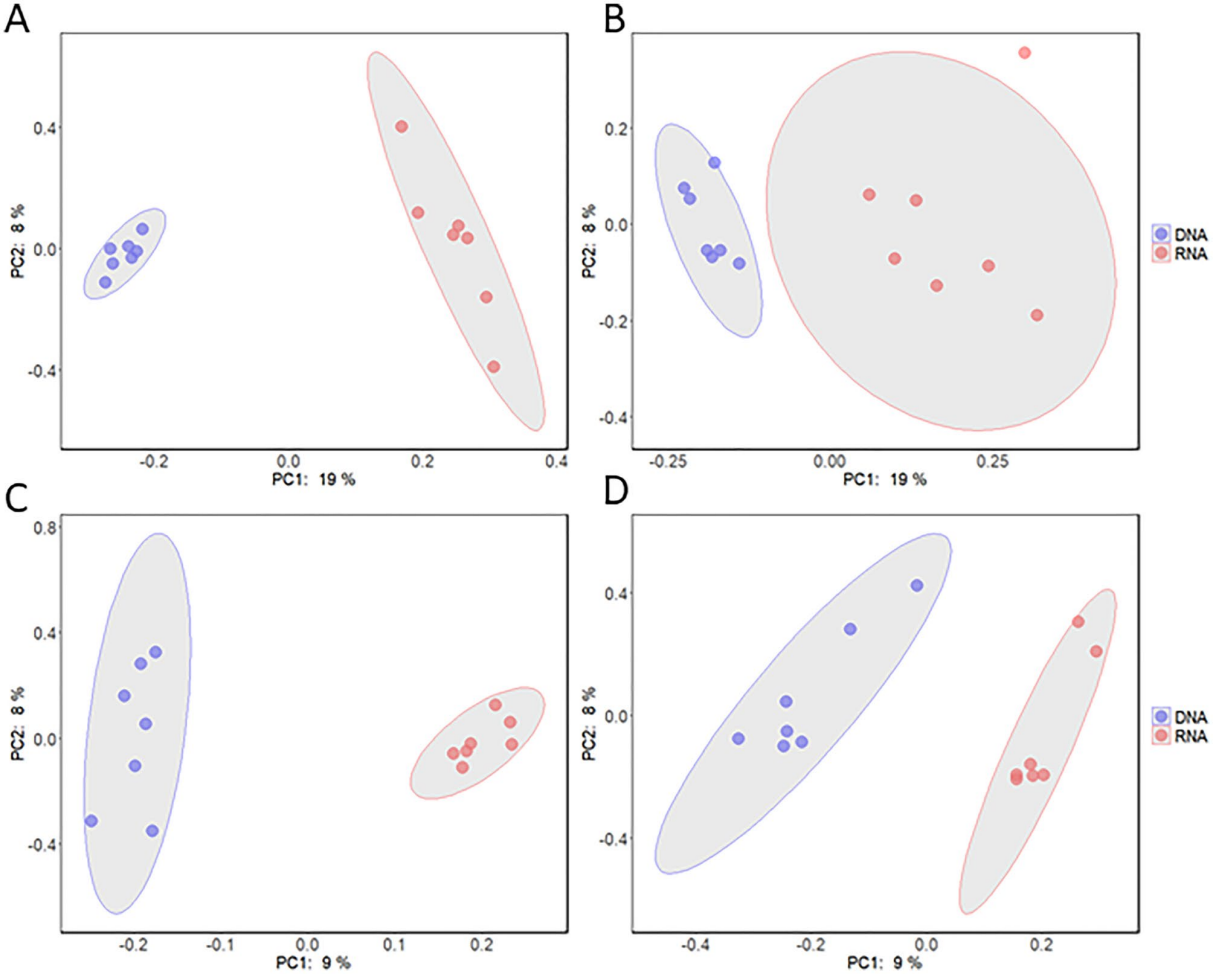
Beta diversity PCOA plots for DNA (teal) vs. RNA (orange) metabarcoding templates for the 18S gene (**A** and **B**) and for the COI gene (**C** and **D**). Bray-Curtis diversity is reported in A and C, and Jaccard’s Distance is reported in B and D. Confidence ellipses were calculated at 95%. All plots show significant differences at *p* < 0.002 in diversity between DNA and RNA template groups using a PERMANOVA analysis (n = 7).

Beta diversity is visualized in Principal Coordinate Analysis plots (PCOA). For the 18S marker, there were significant differences between the clustering of RNA and DNA templates for the Bray–Curtis diversity metric (*p* = 0.002) and for Jaccard’s Distance (*p* = 0.001) (Fig. 5A,B). There were also significant differences between RNA and DNA clustering in the COI marker for both Bray–Curtis diversity (*p* = 0.001) and for Jaccard’s Distance (*p* = 0.002) (Fig. 5C,D).

## Discussion

The current study is the first to directly compare the differences in eukaryotic community diversity by metabarcoding eRNA and eDNA from an estuarine benthic ecosystem. This is also the first study to compare the diversity of environmental RNA and DNA using two of the most common loci examined in metabarcoding applications: COI and 18S. Only a handful of studies have used eRNA to assess diversity through metabarcoding and have demonstrated some differences in detected diversity between eRNA and eDNA in metabarcoding^19,29,38^. Environmental DNA has many useful applications, but one of the downfalls of DNA is that it can be detected long after an organism has inhabited an area, such as from dead organisms and even significant distances away from the source^1^. While the persistence of eDNA may be useful in detecting the presence of endangered, rare, or invasive species in marine systems, the persistence of eDNA, such as legacy DNA, may skew the accurate representation of community structure immediately after a disturbance event or during the exact moment of sampling^21^. Environmental RNA proves to be a useful tool because it is far less persistent in marine environments^2,27^. In the current study, RNA provides a snapshot of living organisms present in the mesocosms at the time of sampling, compared to DNA which detects past and present organisms in the sample.

Benthic ecosystems often have high biodiversity because they are dynamic environments with various substrates favorable for hosting communities^39^. To date, there is no one loci and associated primer pair that will effectively detect all eukaryotic organisms, and in particular metazoans, so it is beneficial to use multiple loci when looking for a variety of taxa^6^. Previous marine eDNA studies have demonstrated the preferred method of using two loci to achieve comprehensive metabarcoding for taxonomically diverse environments^5,30,40^. The current study further demonstrates the need to use at least two different loci for targeted PCR and sequencing to truly capture the wide diversity in rich systems, such as the top oxygenated layer of the marine benthos. Our results demonstrate the types of organisms detected using the 18S loci vary widely from the organisms detected by COI. The 18S loci also detected a higher number of organisms than using the COI loci. The 18S allowed greater detection of metazoans whereas COI was useful in the detection of Oomycota (i.e., a eukaryotic microorganism that resembles fungi) protozoa. Protozoa are often food sources for meiofauna^41^; therefore, using COI for protozoan detection and 18S for metazoan detection showcases multiple trophic levels present in the mesocosms of the current study. Utilizing multiple loci not only broadens taxonomic diversity detection but can also highlight multiple trophic levels for understanding food webs originating in benthic environments^15^.

A study evaluating arctic benthic diversity similarly found that COI detected fewer taxa than 18S using eDNA metabarcoding, and there was only ~ 40% taxa overlap between markers at the class level^42^. In the current study, the COI marker using both nucleic acid templates yielded a higher percentage of unassigned taxa after filtering for presence in the majority of mesocosms compared to 18S. A possible explanation for the low metazoan detection by COI may be that the unassigned taxa are metazoans rather than more SAR organisms. Additionally, singly detected metazoans were filtered out of the analysis if they were not detected in at least 4 mesocosms. For example, one type of amphipod was detected in only 3 mesocosm by RNA using the COI marker, but was not detected in any mesocosms using DNA. Therefore, the amphipod observation was excluded from the COI results in Fig. 3 and Table S3 because it was not found in at least 4 mesocosms.

Overall, RNA provides a broader assessment of benthic community structure than DNA, particularly when using two loci/ markers for sequencing. In the current study, we used nuclear 18S ribosomal RNA and DNA and mitochondrial COI RNA and DNA sequences as markers for metabarcoding. The number of copies of ribosomal RNA per cell is higher than the copies of ribosomal DNA, and the ratio of RNA: DNA is higher in single-cell organisms, such as protists^43^. The higher number of unique ASVs detected using eRNA is likely attributed to the higher number of RNA copies of each marker in small, single-cell organisms successfully amplified during PCR, therefore making rare organisms easier to detect. The DNA of highly abundant or higher biomass organisms may “drown out” (i.e., mask) the sequences from lower abundance and biomass organisms during PCR amplification, thus resulting in lower detected α-diversity. The increased detection of ciliates and protozoa using eRNA are consistent with other recent eRNA metabarcoding results that found higher ciliate and protozoan diversity compared to eDNA using the same Uni18S primers^19^. Positive correlations between organism biomass and sequence copy numbers have been demonstrated for DNA metabarcoding conducted on invertebrate species^44^. In the current study, RNA allowed for the detected of both larger meiofauna and smaller microfauna, which is optimal for assessing true biodiversity with molecular assays. Chaetonotida, a type of gastrotrich, was the only taxa detected in 4 of the mesocosms using DNA that was not found with RNA. It is possible the chaetonotida may have died during the experiment due to sensitivities to new environmental conditions, and therefore were not detected with RNA. The temperature of the flowing seawater in the mesocosms was approximately 18 °C, and many marine chaetonotida prefer 23–28 °C and high organic matter substrate^45^.

Previous studies have compared the accuracy of conventional morphological identification to molecular metabarcoding methods for assessing biodiversity. In estuarine-specific studies, metabarcoding methods are able to detect the majority of taxa identified with traditional methods and often detected higher species richness, or higher numbers of unique organisms, that was not found conventionally^10,46,47^. The limiting factor for higher resolution of taxonomic identification is the availability of species-specific sequences in barcoding databases^48^. However, barcoding databases are becoming more robust as an increasing number of researchers contribute high quality sequencing data to databases^7^. Therefore, eRNA metabarcoding techniques perform similarly to conventional morphological methods, and may even uncover higher biodiversity in systems like estuaries where meiofauna have been historically understudied and identified.

Although eRNA α-diversity is higher compared to eDNA, there is some overlap between ASVs detected with eRNA and eDNA. The higher percentage of overlap between eDNA and eRNA ASVs is predominantly seen in the ASVs detected in all 7 of the mesocosms. The increased overlap in ASVs detected in all 7 mesocosms compared to the relatively lower overlap of ASVs found in only 1 of the 7 mesocosms is due to the filtering of random organisms found in only 1 mesocosm. Detection of a unique organism in a single mesocosm is likely not representative of the sample community and filters potential artifacts that may be introduced during cDNA synthesis from eRNA. However, all uniquely identified ASVs are used in the β-diversity analysis, so the higher number of unique ASVs detected from eRNA are likely driving the significant differences observed between eRNA and eDNA β-diversity. It is possible that using eRNA could increase the statistical power of a study design compared to eDNA because eRNA detects a higher number of ASVs. It is unlikely that a higher number of RNA ASVs could be due to splice variants contributing to unique sequences because the amplified regions of both markers do not contain introns. Therefore, no splicing of transcripts would be expected. Thus, the detected DNA ASVs are from living organisms in the mesocosms and the higher diversity of ASVs detected from RNA demonstrate that RNA is a more suitable option for assessing diversity of living organisms.

It is evident that the mesocosms in the current study were rich with meio- and microfauna due to the number of unique organisms and broad diversity of different taxa. It is likely that collecting samples for nucleic acid extraction directly from the field site may result in higher diversity because there is no mechanical disturbance during the laboratory acclimation period and the presence of other organisms in the system, such as fish, macroinvertebrates, or birds. eDNA molecular abundance in samples has been shown to correlate to actual organismal abundance in laboratory environments, but the same correlation is not as apparent in field samples, which is likely due to a variety of collection and processing methods^49^. eRNA may be the better nucleic acid template for field collection once flash frozen, especially for the detection of protozoans. Future studies will compare the diversity of eukaryotic communities detected using eDNA and eRNA collected directly from the field rather than from sediment core mesocosms. Repeated sampling from a field site may help reduce transient eRNA detection when establishing accurate baseline community composition in field-based biomonitoring studies.

Recently, meiofaunal organisms and communities are being explored as bioindicators, which are organisms whose presence are indicators for environmental stress and pollution^17^. For example, lower meiofaunal diversity and abundance is associated with higher pollution in harbors, and the presence of some genera of nematodes are correlated with higher concentrations of polycyclic aromatic hydrocarbons because they are more tolerant to pollution^50^. A previous study that used field-collected mesocosms from the same location as our current study found similar phyla detected with a metabarcoding approach; the majority of the sequences detected in benthic communities were from nematodes, arthropods, and the microfaunal SAR clade^10^. Similarly, the majority of the ASVs detected from the current study also corresponded with nematodes, arthropods, and the SAR clade, as well as other commonly detected meiobenthic organisms, such as polychaetes and Homalorhagida (i.e., mud dragons). A recent study exposed a benthic foraminiferal community to chromium, and found that eRNA metabarcoding was more robust for detecting changes in diversity at lower chromium concentrations compared to eDNA^51^. The eRNA metabarcoding method used in the current study detected meiofaunal taxa typical of marine or estuarine environments. Therefore, eRNA metabarcoding may be useful for efficiently identifying bioindicator species or taxa impacted by exposures to different contaminants and environmental stressors to aid with management of aquatic systems. Another advantage of eRNA is that RNA provides functional information about how organisms response to stress through altered transcription of activated pathways. eRNA will likely be a more powerful tool than eDNA because it allows for the detection of both bioindicator species and, in the future with increase development of genetic databases, environmental detection of biomarkers of stress through increased transcription of response genes.

Many academic researchers are adopting molecular methods using High Throughput Sequencing as the future of biomonitoring surveys; however, few regulatory and environmental management organizations/ agencies have adopted metabarcoding into their routine biomonitoring practices for regulatory purposes. In marine benthic communities, metabarcoding provides a comprehensive assessment of diversity and is useful for detecting a broader array of organisms in biomonitoring surveys especially when using two markers^47^. eDNA is also useful for monitoring discrete communities (i.e., benthic versus pelagic)^52^, so it is possible that using eRNA could provide vital information about living organisms in specific environments compared to eDNA. Metabarcoding sequencing and bioinformatic approaches for benthic environments vary among studies, thus requiring some standardization between methods to further advance the use of metabarcoding in conservation and regulatory applications^30,53^.

Like eDNA, some advancements must be made with eRNA to be used as a quantitative tool in molecular ecology. Validation of eDNA metabarcoding for assessing relative abundance of species is rapidly progressing by correlating laboratory studies of DNA shedding with field experiments^1,54^. Similar validation techniques can be used to develop eRNA metabarcoding as a quantitative or semi-quantitative method. There is growing interest in optimizing eRNA extraction protocols from different types of environmental media to standardize the use of eRNA in downstream molecular applications^55^. Thus, standardizing eRNA protocols will help with integration into environmental management toolkits for regulatory purposes.

RNA poses unique barcoding challenges compared to DNA because the number of RNA transcripts from a gene are not always present in the same proportion compared to the gene copy number per genome (i.e., one DNA copy per cell), especially for differentially expressed genes. However, one possible way to work around this issue is to utilize constitutively expressed marker loci where transcription is generally stable and unaffected by environmental stressors, such as those used as reference genes for quantitative real-time PCR^49^. Fortunately, many loci chosen for metabarcoding purposes fit this criterium; the transcription of 18S and COI remain steady within the cell regardless of environmental stress.

Collecting sediment cores from the environment and bringing them into controlled laboratory settings for community analysis through eRNA metabarcoding is a powerful tool that opens opportunities for this method to be used in a broad range of fields. For instance, field-collected mesocosms could be used in controlled settings to investigate the effects of individual or mixtures of toxicants on entire community and population-level outcomes. Marine sediments are often the ultimate sink for environmental contaminants, such as organic pollutants , heavy metals^56^, and plastic particles^57^, yet few studies investigate actual community-level changes in contaminant exposures. Marine benthic environments have high biodiversity, but the breadth of diversity in micro- and meiofaunal organisms is often understudied because traditional morphological methods are immensely time consuming. Sediment core mesocosms could also be used to understand the effects of global climate change stressors, such as fluctuating temperatures, surface water salinity and pH, on communities as well as conventional and emerging contaminants in combination with climate change stressors. Additionally, this method could also be used to understand how communities respond to a significant disturbance event or smaller series of stressors, which are often difficult to measure in environmental settings^32,58^. In these applications, eRNA is favorable for constructing community composition at a specific moment of the experiment to better regulate anthropogenic causes of environmental stress.

## Conclusions

eRNA is an efficient, powerful tool for assessing community diversity, particularly when using multiple loci for metabarcoding. While eDNA is a robust tool for capturing all DNA present in a sample, eRNA provides a more comprehensive snapshot of community structure for living organisms within the target community, including protozoans and other microfauna, while eDNA reflects both living and dead organisms. Additionally, eRNA metabarcoding detected higher numbers of unique organisms and greater α-diversity compared to eDNA because eRNA of larger organisms does not “drown out” detection of smaller organisms. The higher detection of microfauna using eRNA may lead to greater statistical power through increased ability to detect significant community differences between treatments. Utilizing eRNA for environmental monitoring can enhance the detection of benthic organisms that are rarely seen through morphological identification. As demonstrated here, combining eRNA metabarcoding with mesocosm studies allows a greater understanding of community and population-level outcomes and can be used as a tool in a plethora of applications where impacts on those levels have been difficult to study. The development of eRNA metabarcoding will ultimately lead to advancements in our knowledge of trophic interactions, ecosystem response to stressors, and taxonomic identification of understudied organisms resulting in more comprehensive management of marine systems. Finally, the ability to accurately reconstruct a microscopic community at a precise moment in time can be improved with the use of eRNA. Metabarcoding approaches, whether using eRNA or eDNA, will only become more robust as primer designs are improved for detection of broader taxa and more species marker sequences are added to databases.

## Supplementary Information

Supplementary Information.

Supplementary Information.

Supplementary Information.

Supplementary Information.

Supplementary Information.

## Data Availability

Sequencing data and metadata files can be accessed through NCBI Sequence Read Archive (SRA) repository as follows: ** Genetic data:** Raw sequence reads are deposited in the SRA (BioProject PRJNA810607). **Sample metadata:** Metadata are also stored in the SRA (BioProject PRJNA810607). https://dataview.ncbi.nlm.nih.gov/object/PRJNA810607?reviewer=38skmg8kl9cn1omiequc0eaale.
