# Supplementary material for: Environmental RNA as a Tool for Marine Community Biodiversity Assessments
Source: Sci Rep. 2022 Oct 22;12:17782. doi: 10.1038/s41598-022-22198-w (PMC9588027; doi:10.1038/s41598-022-22198-w)
Supplement: Supplementary file 1 — Supplementary Information. [file 41598_2022_22198_MOESM1_ESM.docx]

Supplementary Information for:

**Environmental RNA as a Tool for Marine Community Biodiversity Assessments**

^1^ *Marissa Giroux, ^2^ Jay R. Reichman, ^1^ Troy Langknecht, ^3^Robert M. Burgess, ^3^ Kay T. Ho

^1^ORISE c/o U.S. EPA ORD/CEMM Atlantic Coastal Environmental Sciences Division

^2^U.S. EPA, Office of Research and Development, Pacific Ecological Systems Division, Corvallis, OR

^3^U.S. EPA, Office of Research and Development, Atlantic Coastal Environmental Sciences Division, Narragansett, RI

***Corresponding author: Marissa Giroux (giroux.marissa@epa.gov)**


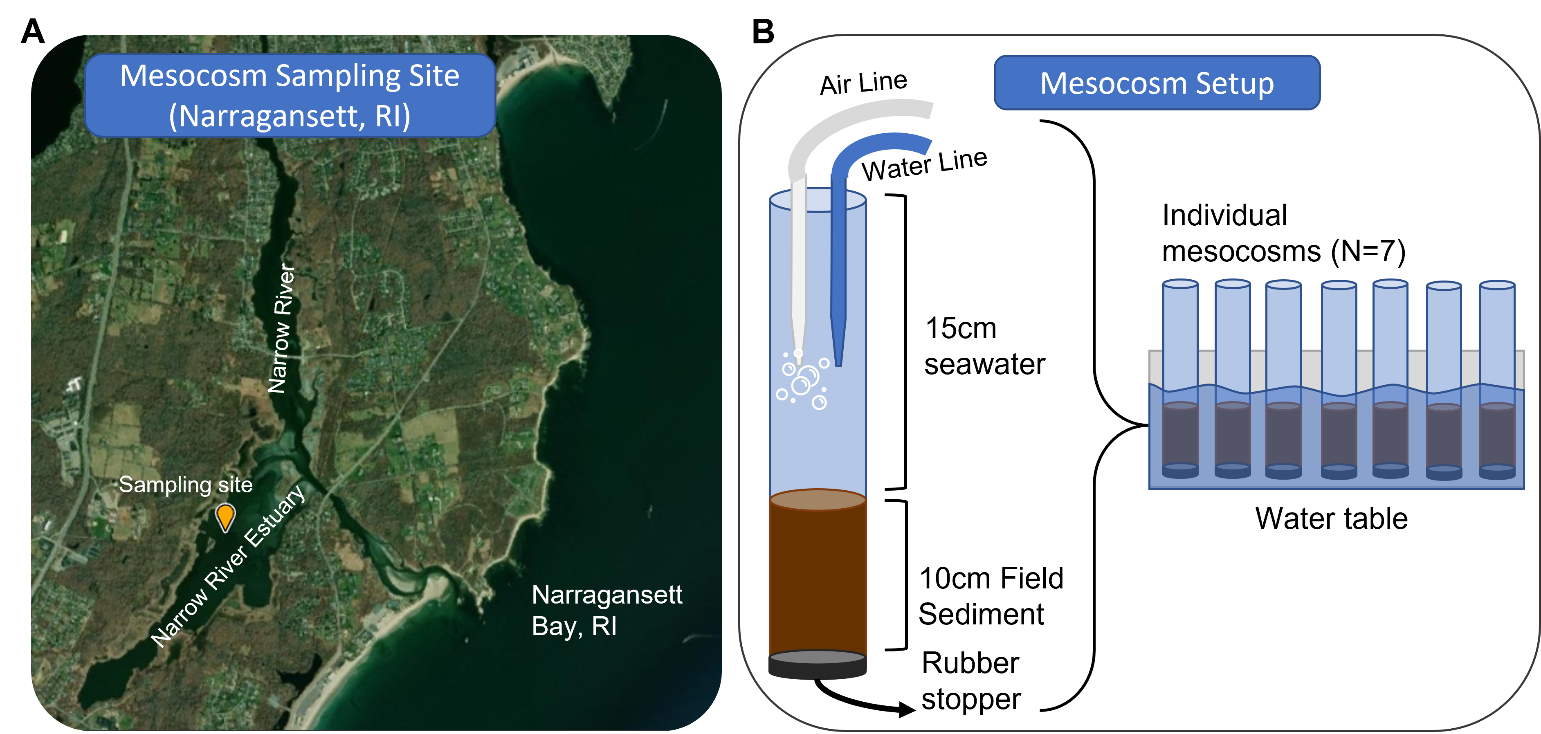


Figure S1, Mesocosm sampling location (A) in Narragansett, RI. The yellow pin represents the exact sampling coordinates at 41° 26.877’ N, -71° 27.528’ W. The laboratory mesocosm setup (B) showcases a diagram of an induvial mesocosm and a diagram of all 7 mesocosms set in a flow-through water table at ambient seawater temperature (18⁰C). The map is sourced from ArcGIS Pro Version 2.8.0 (<https://pro.arcgis.com/en/pro-app/2.8/get-started/install-and-sign-in-to-arcgis-pro.htm>).


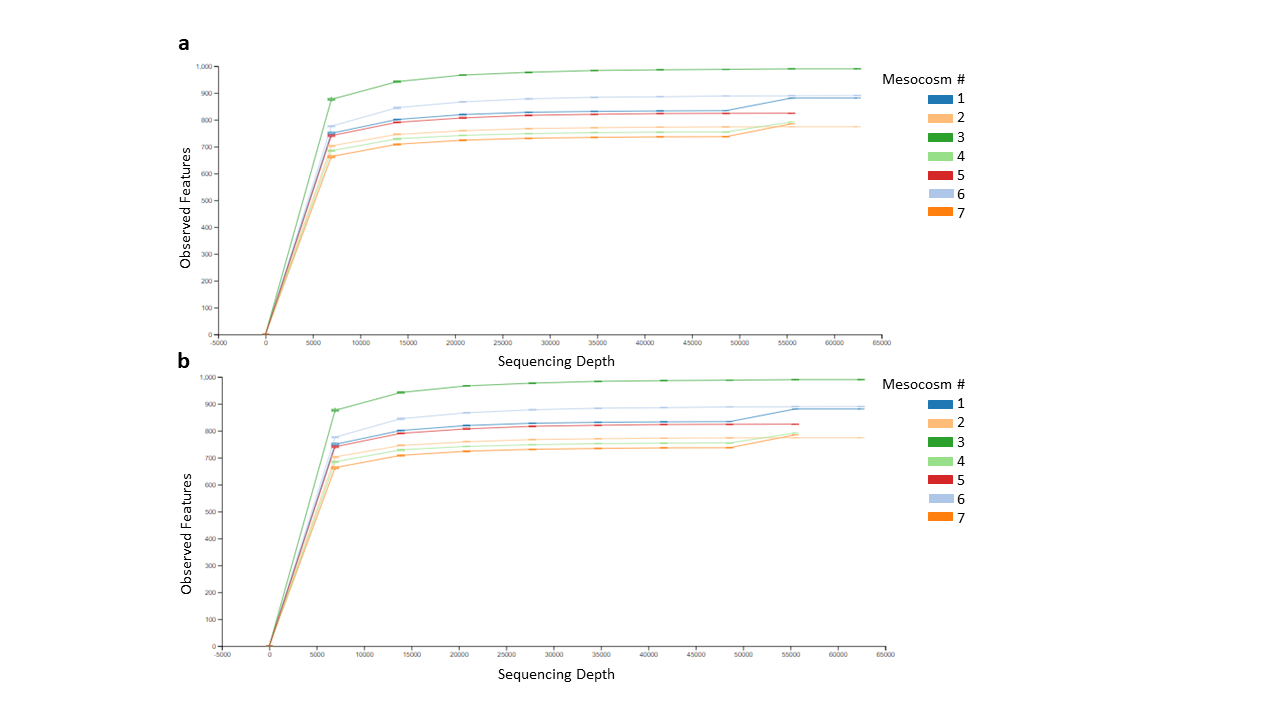


Figure S2, Alpha rarefaction curves for the A) 18S marker and B) COI marker generated after quality control filtering. The y-axis is the number of observed features, and the x-axis is sequencing depth. Each line represents an individual mesocosm with pooled technical replicates.

Figure S3, Relative abundance of all taxa detected in each mesocosm using the 18S marker for both DNA and RNA templates. Taxonomic identification is to the supergroup or Phylum level. There are two technical replicates pooled together for each mesocosm. Each bar represents an individual mesocosm.

Figure S4, Relative abundance of all taxa detected in each mesocosm using the COI marker for both DNA and RNA templates. Taxonomic identification is to the Class level. There are two technical replicates pooled together for each mesocosm. Each bar represents an individual mesocosm.

| SI Table 1. Information on sequencing results for total read number before quality control filtering, and resulting ASV number for individual mesocosms for both COI and 18S markers and RNA and DNA templates | | | | | |
| --- | --- | --- | --- | --- | --- |
| Mesocosm # | Nucleic Acid Template | COI | | 18S | |
|  |  | # Reads | # Features | # Reads | # Features |
| 1 | RNA | 349361 | 65089 | 331262 | 32092 |
| 2 | RNA | 334046 | 63679 | 226636 | 18233 |
| 3 | RNA | 413193 | 83102 | 240726 | 24769 |
| 4 | RNA | 345136 | 56031 | 225976 | 21493 |
| 5 | RNA | 282364 | 62076 | 320117 | 33126 |
| 6 | RNA | 309551 | 78793 | 296450 | 30298 |
| 7 | RNA | 279584 | 61314 | 285172 | 27859 |
| 1 | DNA | 287036 | 55274 | 246864 | 23805 |
| 2 | DNA | 279693 | 62811 | 242147 | 24827 |
| 3 | DNA | 300105 | 64230 | 305140 | 29689 |
| 4 | DNA | 286682 | 51126 | 256770 | 26507 |
| 5 | DNA | 277843 | 57529 | 251141 | 24386 |
| 6 | DNA | 316499 | 70728 | 221074 | 23186 |
| 7 | DNA | 248278 | 50371 | 243201 | 26569 |

| SI Table 2. Taxonomy of unique sequences (ASVs) for the 18S marker for RNA and DNA templates. | | | | | | | | | | |
| --- | --- | --- | --- | --- | --- | --- | --- | --- | --- | --- |
| Group | Class | Order | Number ASVs 4/7 ^a^ | | Number ASVs 7/7 ^b^ | | Percent ASVs 4/7 | | Percent ASVs 7/7 | |
|  |  |  | RNA | DNA | RNA | DNA | RNA | DNA | RNA | DNA |
| Amoebozoa | Discosea |  | 6 | 0 | 0 | 0 | 3% | 0% | 0% | 0% |
| Archaeplastida | Chlorodendrophyceae | Chlorodendrales | 5 | 2 | 3 | 0 | 2% | 1% | 3% | 0% |
| - | - | Ancyromonadida | 1 | 0 | 0 | 0 | 0% | 0% | 0% | 0% |
| - | Apusomonadea | Apusomonadida | 1 | 0 | 0 | 0 | 0% | 0% | 0% | 0% |
| Opisthokonta | Unassigned | - | 2 | 4 | 1 | 4 | 1% | 3% | 1% | 5% |
| Opisthokonta | Choanoflagellatea | Acanthoecida | 1 | 0 | 0 | 0 | 0% | 0% | 0% | 0% |
| Opisthokonta | Polychaeta | Eunicida | 1 | 1 | 0 | 0 | 0% | 1% | 0% | 0% |
| Opisthokonta | Polychaeta | Orbiniidae | 1 | 0 | 0 | 0 | 0% | 0% | 0% | 0% |
| Opisthokonta | Maxillopoda | - | 3 | 3 | 2 | 2 | 1% | 2% | 2% | 3% |
| Opisthokonta | Maxillopoda | Calanoida | 4 | 0 | 0 | 0 | 2% | 0% | 0% | 0% |
| Opisthokonta | Maxillopoda | Cyclopoida | 2 | 6 | 1 | 4 | 1% | 4% | 1% | 5% |
| Opisthokonta | Maxillopoda | Harpacticoida | 12 | 13 | 6 | 7 | 6% | 9% | 7% | 9% |
| Opisthokonta | Ostracoda | Myodocopida | 4 | 3 | 3 | 3 | 2% | 2% | 3% | 4% |
| Opisthokonta | Ostracoda | Podocopida | 4 | 4 | 2 | 2 | 2% | 3% | 2% | 3% |
| Opisthokonta | Gastrotricha | Chaetonotida | 0 | 4 | 0 | 0 | 0% | 3% | 0% | 0% |
| Opisthokonta | Chromadorea | Chromadorida | 25 | 26 | 13 | 19 | 12% | 18% | 14% | 24% |
| Opisthokonta | Chromadorea | Desmodorida | 1 | 1 | 0 | 0 | 0% | 1% | 0% | 0% |
| Opisthokonta | Chromadorea | Monhysterida | 38 | 67 | 26 | 35 | 18% | 47% | 28% | 44% |
| Opisthokonta | Rhabditophora | Rhabdocoela | 1 | 0 | 0 | 0 | 0% | 0% | 0% | 0% |
| Opisthokonta | - | Homalorhagida | 1 | 4 | 0 | 4 | 0% | 3% | 0% | 5% |
| SAR | Unassigned | - | 12 | 4 | 1 | 0 | 6% | 3% | 1% | 0% |
| SAR | Conoidasida | Eucoccidiorida | 1 | 0 | 0 | 0 | 0% | 0% | 0% | 0% |
| SAR | Oligohymenophorea | - | 4 | 0 | 3 | 0 | 2% | 0% | 3% | 0% |
| SAR | Plagiopylea | Plagiopylea | 6 | 0 | 0 | 0 | 3% | 0% | 0% | 0% |
| SAR | Prostomatea | - | 44 | 0 | 16 | 0 | 20% | 0% | 17% | 0% |
| SAR | Litostomatea | - | 4 | 0 | 3 | 0 | 2% | 0% | 3% | 0% |
| SAR | Spirotrichea | - | 17 | 1 | 5 | 0 | 8% | 1% | 5% | 0% |
| SAR | Karyorelictea | Protostomatida | 3 | 0 | 0 | 0 | 1% | 0% | 0% | 0% |
| SAR | Dinophyceae | - | 5 | 1 | 0 | 0 | 2% | 1% | 0% | 0% |
| SAR | Chromerida | - | 3 | 0 | 3 | 0 | 1% | 0% | 3% | 0% |
| SAR | Chlorarachniophyta | Minorisida | 1 | 0 | 0 | 0 | 0% | 0% | 0% | 0% |
| SAR | Imbricatea | Euglyphida | 3 | 0 | 0 | 0 | 1% | 0% | 0% | 0% |
| SAR | Thecofilosea | Cryomonadida | 4 | 0 | 4 | 0 | 2% | 0% | 4% | 0% |
|  |  | **Total** | **220** | **144** | **92** | **80** |  |  |  |  |
| ^a^ Number of ASVs detected in at least 4 of the 7 mesocosms  ^b^ Number of ASVs detected in all 7 mesocosms | | | | | | | | | | |

| SI Table 3. Taxonomy of unique sequences (ASVs) for the COI marker for RNA and DNA templates. | | | | | | | | | |
| --- | --- | --- | --- | --- | --- | --- | --- | --- | --- |
| Class | Order | Number ASVs 57% **^a^** | | Number ASVs 100%**^b^** | | Percent ASVs 57% | | Percent ASVs 100% | |
|  |  | RNA | DNA | RNA | DNA | RNA | DNA | RNA | DNA |
| Oomycota | Pythiales | 36 | 10 | 10 | 1 | 28% | 17% | 27% | 6% |
| Oomycota | Saprolegniales | 12 | 2 | 2 | 0 | 9% | 3% | 5% | 0% |
| Oomycota | Peronosporales | 0 | 2 | 0 | 0 | 0% | 3% | 0% | 0% |
| Oomycota | Unassigned | 72 | 36 | 21 | 10 | 56% | 60% | 57% | 63% |
| Gastrotricha | Chaetonotida | 3 | 3 | 3 | 3 | 2% | 5% | 8% | 19% |
| Chromadorea | Monhysterida | 1 | 0 | 1 | 0 | 1% | 0% | 3% | 0% |
| Hexanauplia | Harpacticoida | 0 | 2 | 0 | 0 | 0% | 3% | 0% | 0% |
| Unassigned | - | 4 | 5 | 0 | 2 | 3% | 8% | 0% | 13% |
|  | **Total** | **129** | **60** | **37** | **16** |  |  |  |  |
| ^a^ Number of ASVs detected in at least 4 of the 7 mesocosms  ^b^ Number of ASVs detected in all 7 mesocosms | | | | | | | | | |
