# Supplementary material for: Environmental RNA as a Tool for Marine Community Biodiversity Assessments
Source: Sci Rep. 2022 Oct 22;12:17782. doi: 10.1038/s41598-022-22198-w (PMC9588027; doi:10.1038/s41598-022-22198-w)
Supplement: Supplementary file 4 — Supplementary Information. [file 41598_2022_22198_MOESM4_ESM.qzv › 17cca31a-1607-434d-b612-8e8b20d85cdb/data/index.html]

q2\_metadata : tabulate


Download metadata TSV file

This file won't necessarily reflect dynamic sorting or filtering
options based on the interactive table below.
